# Floating body effect in indium–gallium–zinc–oxide (IGZO) thin-film transistor (TFT)

**DOI:** 10.1038/s41598-024-60288-z

**Published:** 2024-05-02

**Authors:** Jingyu Park, Seungwon Go, Woojun Chae, Chang Il Ryoo, Changwook Kim, Hyungju Noh, Seonggeun Kim, Byung Du Ahn, In-Tak Cho, Pil Sang Yun, Jong Uk Bae, Yoo Seok Park, Sangwan Kim, Dae Hwan Kim

**Affiliations:** 1 School of Electrical Engineering, Kookmin University, Seoul, 02707 Republic of Korea; 2 Department of Electronic Engineering, Sogang University, Seoul, 04107 Republic of Korea; 3 Large Display Business Unit, LG Display Company, Paju, 10845 Republic of Korea

**Keywords:** Engineering, Materials science, Nanoscience and technology

## Abstract

In this paper, the floating body effect (FBE) in indium-gallium-zinc-oxide (IGZO) thin-film transistor (TFT) and the mechanism of device failure caused by that are reported for the first time. If the toggle AC pulses are applied to the gate and drain simultaneously for the switching operation, the drain current of IGZO TFT increases dramatically and cannot show the on/off switching characteristics. This phenomenon was not reported before, and our study reveals that the main cause is the formation of a conductive path between the source and drain: short failure. It is attributed in part to the donor creation at the drain region during the high voltage (*V*_high_) condition and in part to the donor creation at the source region during the falling edge and low voltage (*V*_low_) conditions. Donor creation is attributed to the peroxide formation in the IGZO layer induced by the electrons under the high lateral field. Because the donor creation features positive charges, it lowers the threshold voltage of IGZO TFT. In detail, during the *V*_high_ condition, the donor creation is generated by accumulated electrons with a high lateral field at the drain region. On the other hand, the floating electrons remaining at the short falling edge (i.e., FBE of the IGZO TFT) are affected by the high lateral field at the source region during the *V*_low_ condition. As a result, the donor creation is generated at the source region. Therefore, the short failure occurs because the donor creations are generated and expanded to channel from the drain and source region as the AC stress accumulates. In summary, the FBE in IGZO TFT is reported, and its effect on the electrical characteristics of IGZO TFT (i.e., the short failure) is rigorously analyzed for the first time.

## Introduction

An amorphous indium-gallium-zinc-oxide (IGZO) thin-film transistor (TFT) has been widely used in the field of high-performance display and complementary metal–oxide–semiconductor (CMOS) back-end-of-line (BEOL) circuits due to its high mobility (> 10 cm^2^/V s), ultra-low leakage current, large on/off current ratio, large-area uniformity, low cost and low-temperature process^1–7^. Despite these advantages, the IGZO TFT has suffered from several technical issues, such as the development of p-type semiconductor^8^, bias instability (i.e., DC and AC stresses)^9–16^ and reliability problems related to the oxygen vacancy^17,18^, excessive oxygen^19,20^, and metal cation^21^. Therefore, many research groups have been studied for the reliability of IGZO TFT^8–21^.

In this study, the floating body effect (FBE) in IGZO TFT and device failure due to FBE are reported for the first time. In addition, the mechanism and physics are compared with the FBE in silicon-on-insulator (SOI) metal–oxide–semiconductor field-effect transistor (MOSFET)^22–24^. In the case of n-channel SOI MOSFET, the electron–hole pairs (EHPs) are generated by impact ionization as the high electric field is induced at channel-drain junction during the saturation mode [i.e., the high gate voltage (*V*_GS_) and drain voltage (*V*_DS_)]. The electrons can move toward the drain electrode while the holes are accumulated at the floating body. The accumulated holes increase the body potential, which lowers the threshold voltage (*V*_th_) and increases the drain current (*I*_D_). Therefore, the stored holes at the floating body cause the degradation of the device and/or circuit reliability, such as the history effect, propagation delay, and so on^22–27^.

On the other hand, it is well known that FBE rarely occurs in the IGZO TFT because it features intrinsic n-type and low impact ionization generation rate due to a large bandgap (*E*_g_ > 3 eV)^28–30^. However, the IGZO TFT is mainly used for the gate driver in display applications, which requires a higher supply voltage than CMOS logic. Furthermore, it is vulnerable to FBE regarding circuit topology because the driver usually uses a gated-diode structure (i.e., synchronized gate and drain)^31–34^.

In this study, the FBE in IGZO TFT, which occurs when the AC pulse is applied for gated-diode operation, is reported for the first time, and the mechanism is analyzed. This paper is organized as follows. First, the fabricated device structure and measurement method are explained. Next, the electrical characteristics of IGZO TFTs are demonstrated, and their degradation mechanism due to FBE is proposed. After the mechanism is examined by the technology computer-aided design (TCAD) simulation, the FBE in IGZO TFT is compared with that in SOI MOSFET for precise analysis.

## Device fabrication and measurement method

**Figure 1:**
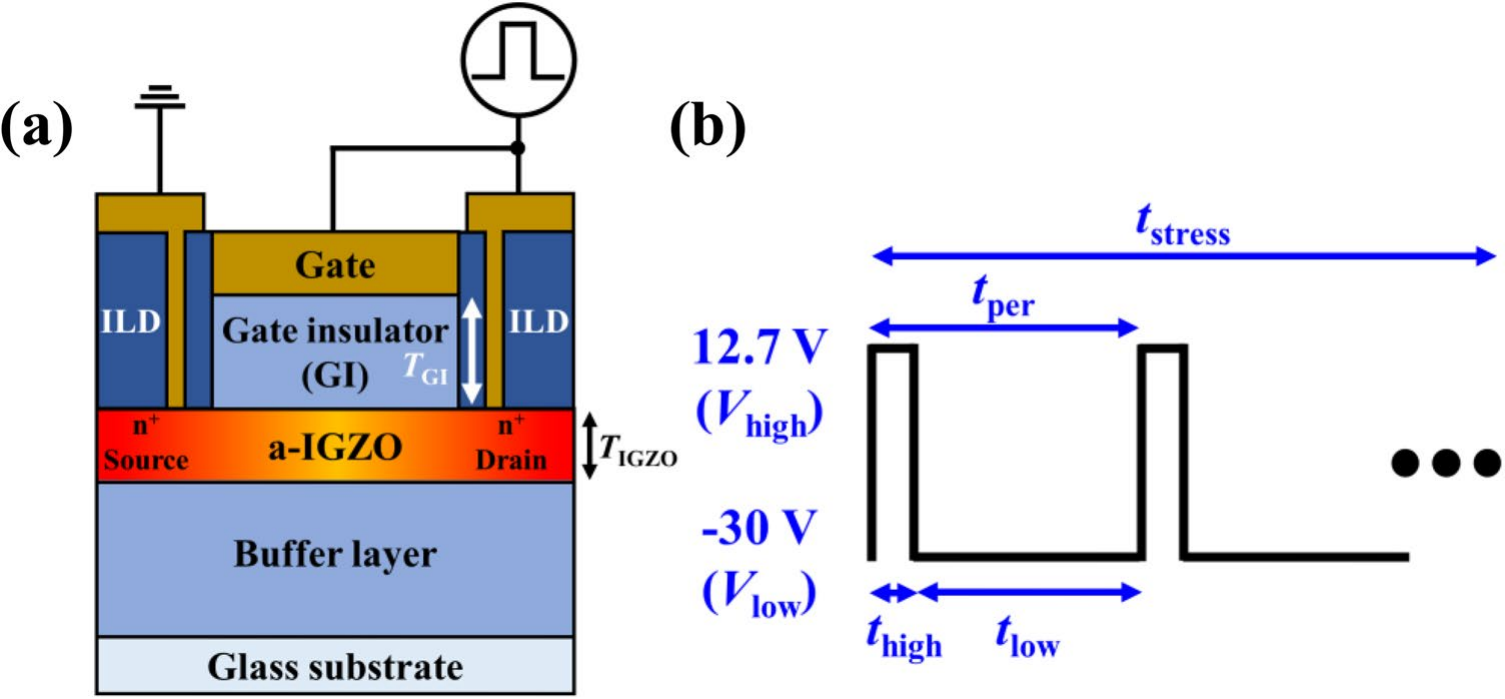
Schematics of (a) IGZO TFT and (b) synchronized gate and drain AC pulse.

Figure 1a shows the schematic of the IGZO TFT with a self-aligned top gate structure. The channel length and width were 6 and 250 µm, respectively. After depositing a 300 nm-thick SiO_2_ buffer layer on the glass substrate by using plasma-enhanced chemical vapor deposition (PECVD), a 30 nm-thick amorphous IGZO channel (In:Ga:Zn = 1:1:1 mol%) was deposited by DC sputtering. The 150 nm-thick SiO_2_ gate oxide and Cu/MoTi gate were deposited by PECVD and DC sputtering, respectively. After gate patterning, the plasma treatment was performed for the highly conductive source/drain region^35^. Subsequently, the interlayer dielectric (ILD) was deposited and patterned for the source/drain region. The source/drain electrodes were formed by Cu/MoTi.

The gated diode (i.e., synchronized gate and drain) IGZO TFT is usually used as a switching device in the gate driver circuit and transmits the image signal to the pixel circuit. Therefore, the electrical characteristics were investigated after applying toggle pulses to examine the switching application. As shown in Fig. 1b, the switching pulses were composed of 12.7 V-high voltage (*V*_high_) and − 30 V-low voltage (*V*_low_) and applied to the gate and drain simultaneously (Fig. 1a). In addition, the pulse was set to 16.6% duty cycle with 30 ms period (i.e., the pulse with 5 ms of *V*_high_ and 25 ms of *V*_low_) and 100 ns of rising/falling time.

## Results and discussion

### Electrical characteristics and degradation mechanism of IGZO TFT with AC pulse

**Figure 2:**
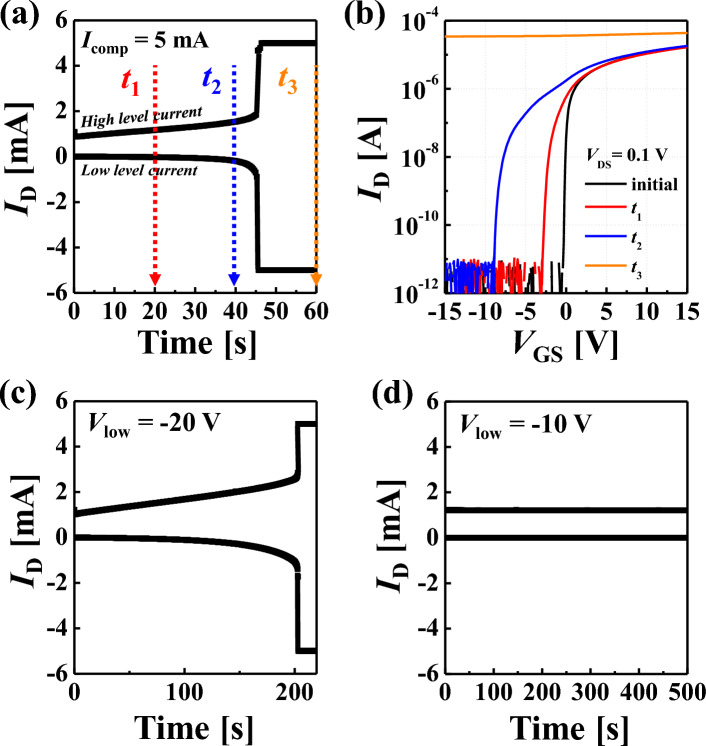
(**a**) Transient response of drain current (*I*_*D*_) under the AC stress shown in Fig. 1b (i.e., *V*_high_ = 12.7 V and *V*_low_ = − 30 V). Here, the high level and low level current are measured during *V*_high_ and *V*_low_, respectively. (**b**) Transfer curves before and after AC stress pulses are applied to the IGZO TFT. Transient response of *I*_*D*_ under the AC stress with the different *V*_low_ from (**a**); (**c**) *V*_low_ = − 20 V and (**d**) *V*_low_ = − 10 V.

Figure 2a shows the drain current (*I*_*D*_) under the AC pulse stress in Fig. 1b. Because a period of pulse is 30 ms, the stress time 20 s (*t*_1_), 40 s (*t*_2_), and 60 s (*t*_3_) are corresponded to 666, 1333, and 2000 pulse stress, respectively. As the stress time increases, the *I*_D_ increases gradually. It is attributed to the decrease of *V*_th_ (Fig. 2b). Generally, these phenomena can be explained by the donor creation at the channel adjacent to the drain region^11,12,36^. The origin of donor creation in IGZO is well known as the formation of peroxide (i.e., O^2−^ + O^2−^ → O_2_^2−^ + 2e^−^) when the strong electric field is applied to the large amount of electrons^19,20^. In detail, if the IGZO TFT is fully turned on (i.e., strong accumulation at *V*_GS_ = *V*_high_) and large *V*_DS_ is applied, the high lateral electric field is applied at the channel-drain junction with high electron concentration at the channel. It can be confirmed that the extracted subgap density-of-state (DOS) [g(e)] increases after stress, corresponding to the generation of donor creation, as shown in Figure S1. As a result, there are donor creations, and the increment of carrier concentration lowers *V*_th_.

The interesting point is that the *I*_D_ rapidly increases to the compliance current after 45 s (Fig. 2a) and cannot show on/off switching characteristics (Fig. 2b, *t*_3_). The detailed images for the short failure are described in the supplementary information (including Figure S2). It is analyzed by the short failure between the source and drain, not the gate leakage current (Figure S3). However, the short failure cannot be explained by the abovementioned donor creation because it is locally generated at the channel adjacent to the drain. In addition, the degradation is affected by the amplitude of *V*_low_, as shown in Fig. 2a, c, and d. In detail, the short failure occurs after 45 s and 200 s with − 30 V and − 20 V-*V*_low_, respectively, while there is no failure with − 10 V-*V*_low_ until 500 s stress time. In other words, the required number of stress pulses (i.e., the stress time) is increased with the smaller *V*_low_. However, the result cannot be explained by the donor creation at the drain region because the electric field at the drain is rarely affected by the amplitude of *V*_low_. Therefore, a novel degradation mechanism is needed to explain these phenomena: short failure and its dependence on *V*_low_.

**Figure 3:**
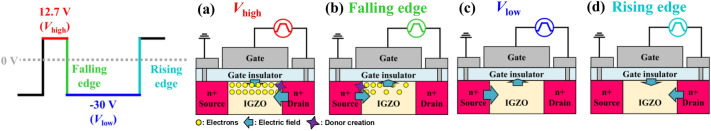
Degradation mechanism of IGZO TFT during AC pulse stress under (**a**) *V*_high_, (**b**) falling edge, (**c**) *V*_low_, and (**d**) rising edge conditions.

Our group hypothesizes that there are different degradation mechanisms depending on the segment of AC pulse applied to the gate and drain simultaneously (Fig. 3). First, in the case of *V*_high_ (Fig. 3a), the electrons at the channel are affected by a high lateral electric field, especially around the drain region due to the large *V*_DS_. As a result, the donor creation occurs at the drain region, as discussed before. Second, a number of accumulated electrons remains without recombination during the 100 ns falling edge (i.e., the transition from *V*_high_ to *V*_low_) because the lifetime of electrons in IGZO is ~ μs^37–39^. At the same time, the electric field at the source is significantly increased when the applied voltage decreases from 0 V to *V*_low_. As a result, the donor creation occurs at the source region since the remaining electrons at the floating body are affected by the strong lateral electric field at the source region. This phenomenon (i.e., the donor creation at the source during the falling edge) is defined as the FBE in IGZO TFT and analyzed in detail (will be discussed later). Third, in the *V*_low_ condition (Fig. 3c), although the lateral electric field is high enough, there is no donor creation because most of the electrons are recombined. Similarly, there are not enough electrons during the rising edge (i.e., the transition from *V*_low_ to *V*_high_) for the donor creation (Fig. 3d). In summary, if the gated-diode operation is repeated (i.e., synchronized AC pulses are applied to the gate and drain repeatedly), the donor creation occurs at the drain during the *V*_high_ condition due to the accumulated electrons. In contrast, the FBE-induced donor creation occurs at the source during the falling edge due to the floating electrons.

**Figure 4:**
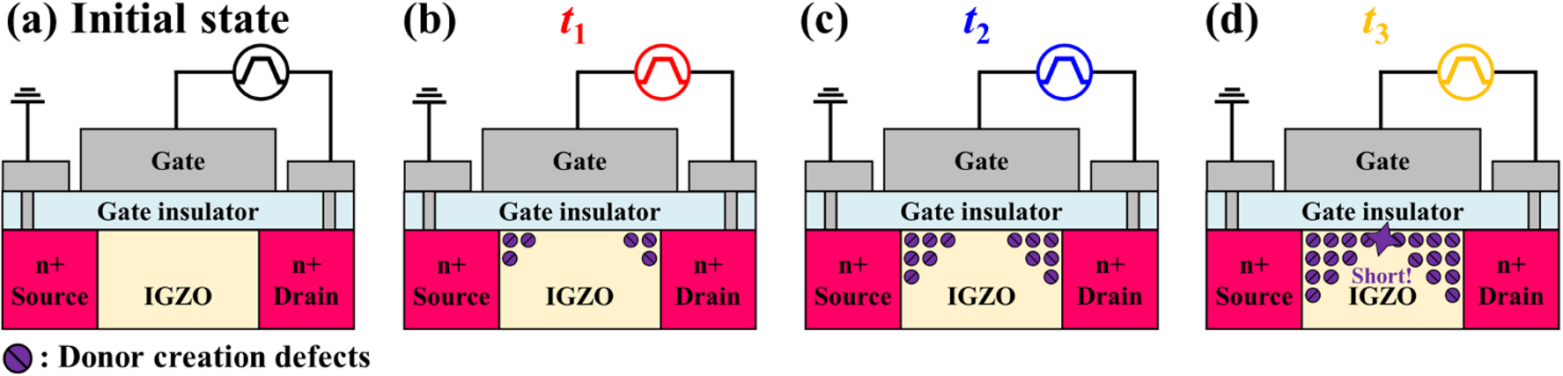
Schematic of short fail mechanism during ac stress with (**a**) initial condition and after (**b**) *t*_1_, (**c**) *t*_2_, and (**d**) *t*_3_ in Fig. 2a.

Figure 4 shows the short failure steps in IGZO TFT (Fig. 2b, *t*_3_) according to the number of the applied AC pulses. As mentioned above, the donor creation is generated at the drain and source during *V*_high_ (Fig. 3a) and falling edge (Fig. 3b), respectively. Moreover, the donor creation is accumulated as the number of AC pulses increases. In the early stage of stress (Fig. 4b), the *V*_th_ is decreased due to the increase of channel potential (Fig. 2b, *t*_1_). As the number of applied pulses increases, the donor creation region gradually expands from the drain and source to the channel, resulting in a decrease in the effective channel length (Fig. 4c). Finally, as shown in Fig. 4d, the donor creation occurs in most channel regions. It results in short failure (channel cannot be OFF despite *V*_low_), and the switching application of IGZO TFT is impossible (Fig. 2a and b, *t*_4_). It is clear that the phenomena, which cannot be explained by the donor creation at the drain region, can be well explained by the proposed FBE-induced donor creation (for evidence of the generation of the donor creation at the drain/source region and the short failure, see the Supplementary Information, Figure S4). The critical point of the proposed degradation mechanism is that the electrons cannot be recombined during the falling edge (i.e., the FBE in IGZO TFT).

### TCAD simulation of the FBE in IGZO TFT

**Figure 5:**
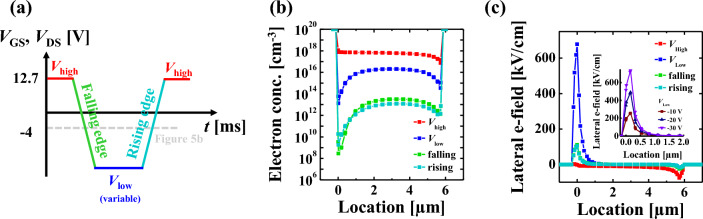
(**a**) Mixed-mode TCAD simulation pulse setup, (**b**) electron concentration distribution with − 30 V-*V*_low_ and (**c**) electric field distribution at *V*_high_, *V*_low,_ and falling/rising edges in channel region along to source-drain direction. The inset of (**c**) shows the electric field with the different *V*_low,_ which corresponds to Fig. 2a,b, and c; − 30 V (purple), − 20 V (navy), − 10 V (wine).

The mixed-mode TCAD simulation is performed to verify the proposed degradation mechanism, the FBE in IGZO TFT. The parameter of DOS is extracted and adapted to the IGZO layer for precise simulation (Table S1). More details about the TCAD simulation are described in the supplementary. The AC pulse is set as in Fig. 5a, and the distribution of electron concentration and electric field along the channel are extracted at *V*_high_, *V*_low_, falling, and rising edges. The falling and rising edges are defined when the AC pulse is − 4 V as shown in the gray dot line in Fig. 5a. As shown in Fig. 5b, the electron concentration at the falling edge and at the rising edge, the former is much larger than the latter. In other words, it is confirmed a large number of floating electrons remain without recombination during the falling edge. At the same time, Fig. 5c shows the electric field at the source region is significant in order of the *V*_low_, falling/rising edges, and *V*_high_. As a result, during the falling edge, the floating electrons at the channel are affected by the high lateral electric field, generating the donor creation at the channel adjacent to the source region. Furthermore, the inset of Fig. 5c shows that the electric field decreases as the *V*_low_ lowers, which is well corresponds to the tendency of short failure with the various amplitudes of the *V*_low_ (Fig. 2a,c, and d). In conclusion, the short failure in IGZO TFT is attributed in part to the donor creation at the drain region during *V*_high_ and in part to the FBE-induced donor creation at the source region during the falling edge.

### Comparison between the FBEs in IGZO TFT and SOI MOSFET

**Figure 6:**
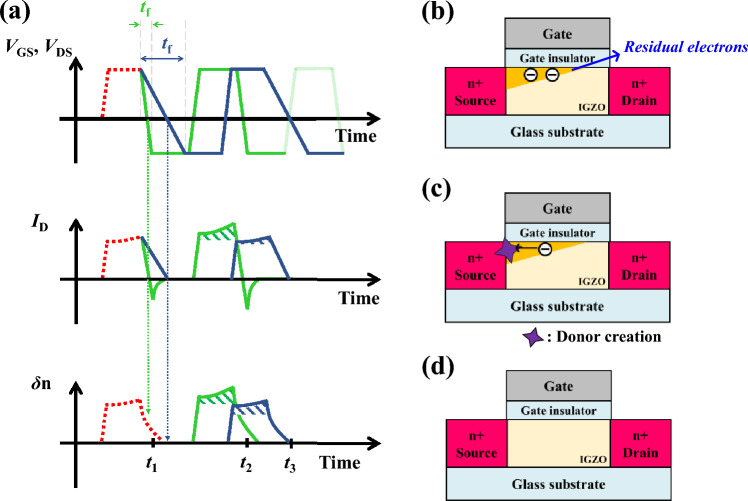
The schematics of (**a**) bias condition (*V*_GS_ and *V*_DS_), *I*_D_, and electron concentration of IGZO TFT with initial pulse and the next pulse applied with short falling time (green line) and long falling time (indigo line), (**b**) FBE in IGZO TFT at the *t*_1_ (i.e., *V*_GS_ and *V*_DS_ change from *V*_high_ to 0 V; *t*_f,high_) which occurs regardless of falling time. The schematics of IGZO TFT during the (**c**) short falling time (*t*_2_) and (**d**) long falling time (*t*_3_).

In this section, the FBEs in IGZO TFT and SOI MOSFET are compared for precise analysis. Figure 6a shows the schematics of *V*_GS_, *V*_DS_, *I*_D_, and electron concentration of IGZO TFT with the initial and subsequent AC pulses. As discussed in the previous section, the FBE-induced donor creation can be generated if the high lateral electric field is applied to the floating electrons, which are generated at *V*_GS_ = *V*_DS_ = *V*_high_ and cannot be recombined during the falling edge because their lifetime is more prolonged than *V*_high_ to *V*_low_ transition time (Fig. 6b; FBE in IGZO TFT). Therefore, the transient time from *V*_high_ to *V*_low_ at the falling edge and the amplitude of the *V*_low_ (i.e., lateral electric field) are the most significant factors for FBE in IGZO TFT. In the case of short *t*_f_ (green line), there is donor creation because the floating electrons are affected by the high lateral electric field (Fig. 6c). The donor creation makes *V*_th_ decrease (Fig. 2b), and hence, the *I*_D_ during the following pulse is more significant than that for the initial pulse. As a result, more floating electrons are generated/accumulated in the channel, and FBE is accelerated. On the contrary, if the falling edge time (*t*_f_) is longer than the electron lifetime (indigo line, Fig. 6d), the FBE-induced donor creation cannot be generated because most of the electrons are recombined, and hence, there are not enough electrons when the source lateral electric field is increased (i.e., *V*_GS_ and *V*_DS_ change from 0 V to *V*_low_; *t*_f,low_). Therefore, it shows the same results for the initial and following pulses regarding *I*_*D*_ and electron concentration.

**Figure 7:**
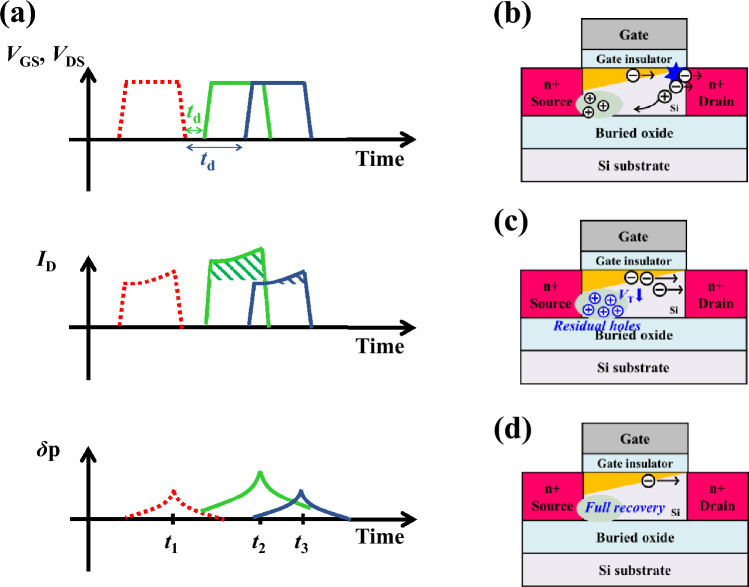
The schematics of (**a**) bias condition (*V*_GS_ and *V*_DS_), *I*_D_, and hole concentration of SOI MOSFET with the initial pulse and the next pulse applied with short delay time (green line) and long delay time (indigo line), (**b**) FBE in SOI MOSFET at the *t*_1_ which occurs regardless of delay time. The schematics of SOI MOSFET during the (**c**) short delay time (*t*_2_) and (**d**) long delay time (*t*_3_).

On the other hand, as shown in Fig. 7a, the FBE in SOI MOSFET is defined as the decrease of *V*_th_ due to the accumulated holes at the floating body in the saturation operation (i.e., *V*_high_). Compared with FBE in IGZO TFT, the accumulation of carriers in the floating body is very similar (Fig. 7b). However, unlike the IGZO TFT, the accumulated holes in SOI MOSFET directly influence the device performance, *V*_th_ shift. Therefore, the FBE is more affected by the delay time (*t*_d_; the time between the initial pulse and the following pulse), which determines the recombination rate of the floating holes rather than *t*_f_. In the case of short *t*_d_ (green line, Fig. 7c), the overdrive voltage (i.e., *V*_GS_-*V*_th_) increases during the following pulse since the floating holes lower *V*_th_. As a result, despite the same bias condition, the larger *I*_*D*_ generates larger excess holes due to impact ionization, and there is positive feedback regarding *I*_*D*_ and floating holes. In contrast, if *t*_d_ is longer than the lifetime of floating holes (indigo line, Fig. 7d), the SOI MOSFET under the following pulse is in the same state as that under the initial pulse because all floating holes are recombined.

Consequently, the FBEs in IGZO TFT and SOI MOSFET are similar because the accumulated carriers in the floating body influence the following pulse in both cases. However, there are main differences in terms of the way that the floating carriers impact the device characteristics. In detail, the floating electrons in IGZO TFT induce the donor creation if there is a sufficient lateral electric field. At the same time, the floating holes directly change body potential and *V*_th_ in SOI MOSFET. Therefore, the dominant factors for FBEs are *t*_d_ in SOI MOSFET and *t*_f_ in IGZO TFT, respectively. It is noteworthy that the energy bandgap of IGZO is about three times larger than that of Si. Therefore, there are a tiny number of holes in IGZO, and the electron lifetime is much longer than that for Si. In addition, the FBE in SOI MOSFET can be suppressed by adjusting the doping concentration and by using trap engineering^40–44^. However, in the case of IGZO TFT, only a limited range of doping concentration is allowed^45,46^, and trap engineering is complex to use due to the amorphous active film structure^47,48^. In conclusion, even though IGZO TFT is more robust than SOI MOSFET in impact ionization, it can be more vulnerable to FBE depending on circuit operating conditions and results in short failure.

## Conclusion

In this study, the FBE in IGZO TFT is reported for the first time, and the effect on the device characteristics is investigated. If the AC pulse is applied to the gate and drain simultaneously for switching operation, the donor creation at drain region during the *V*_high_ condition and the FBE-induced donor creation at the source region should be considered during the falling edge. In detail, the floating electrons at the channel adjacent to the source region are accelerated and activate the peroxide formation if the transition time from *V*_high_ to *V*_low_ is fast enough. Therefore, the short failure occurs as the AC pulse is applied to the gated diode IGZO TFT (i.e., synchronized gate and drain) because the donor creation at the drain and source regions are generated simultaneously and expanded to the channel. Therefore, the FBE reported for the first time in this manuscript must be considered for reliable signal transmission between the gate driver circuits and pixel circuits.

### Supplementary Information

Supplementary Information.

## Data Availability

The datasets used and/or analysed during the current study available from the corresponding author on reasonable request.
