# Supplementary material for: Floating body effect in indium–gallium–zinc–oxide (IGZO) thin-film transistor (TFT)
Source: Sci Rep. 2024 May 2;14:10067. doi: 10.1038/s41598-024-60288-z (PMC11066109; doi:10.1038/s41598-024-60288-z)
Supplement: Supplementary file 1 — Supplementary Information. [file 41598_2024_60288_MOESM1_ESM.docx]

Supplementary Information

**Floating body effect in Indium-Gallium-Zinc-Oxide (IGZO) thin-film transistor (TFT)**

# Jingyu Park^‡,^1, Seungwon Go^‡,^2, Woojun Chae3, Chang Il Ryoo3, Changwook Kim1, Hyungju Noh2, Seonggeun Kim2, Byung Du Ahn3, In-Tak Cho3, Pil Sang Yun3, Jong Uk Bae3, Yoo Seok Park3, Sangwan Kim2,*, and Dae Hwan Kim1,**

1School of Electrical Engineering, Kookmin University, Seoul, 02707, Republic of Korea.

2Department of Electronic Engineering, Sogang University, Seoul, 04107, Republic of Korea.

3Large Display Business Unit, LG Display Company, Paju, 10845, Republic of Korea.

*sangwan@sogang.ac.kr

**drlife@kookmin.ac.kr

^‡^ These authors contributed equally.

***CV* measurement and density of states extraction.** Figure S1a shows an image for measuring photo-response capacitance-voltage (*CV*) characteristics. The wavelength and power of monochromatic light are 532 nm (i.e., photon energy = 2.33 eV) and 5 mW, respectively. Figure S1b shows the *CV* characteristics under the dark and illumination with 100 kHz frequency. It clearly shows that the difference between capacitance under the dark and illumination increases after stress (*t*_1_, 20 sec) while the difference is negligible at the initial state. This implies the generation of donor creation during the AC pulse stress. Using the *CV* curves (figure S1b), the subgap density of states (DOS) is extracted by the single-scan monochromatic phonic capacitance-voltage (MPCV) technique (figure S1c)^S1^. The energy distribution of the acceptor-like DOS and donor-like DOS can be represented by the following equations, respectively.

$$g_{A}\left( E \right)=g_{TA}\left( E \right)+g_{DA}\left( E \right)=N_{TA}\exp\left( -\frac{E_{C}-E}{kT_{TA}} \right)+N_{DA}\exp\left( -\frac{E_{C}-E}{kT_{DA}} \right) (S1)$$

$$g_{D}\left( E \right)=g_{TD}\left( E \right)+g_{SD}\left( E \right){=N}_{TD}\exp\left( -\frac{E-E_{V}}{kT_{TD}} \right)+N_{SD}\exp\left( -\left( \frac{E_{C}-E_{SD}-E}{kT_{SD}} \right)^{2} \right) (S2)$$

Here, the *g*_TA_(E), *g*_DA_(E) are the acceptor-like tail and deep states, respectively, and *g*_TD_(E) [not shown in figure S1(c)] and *g*_SD_(E) are the donor-like tail and shallow states, respectively. The *N*_TA_/*N*_DA_/*N*_TD_/*N*_SD_ parameters are state concentration per energy (cm^-3^eV^-1^), *kT*_TA_/*kT*_DA_/*kT*_TD_/*kT*_SD_ parameters are characteristic energy (eV) that determines the slope of an exponential distribution or width of a Gaussian distribution. The *E*_SD_ parameters are the center of the Gaussian distribution, *E*_C_ and *E*_V_ are the conduction and valance band edge energy, respectively. The extracted parameters are summarized in Table S1. The noteworthy point is that the donor-like state [i.e., *g*_SD_(E)] near the *E*_c_ generated by the donor-like creation increases noticeably compared to the acceptor-like states [i.e., *g*_TA_(E) and *g*_DA_(E)]^S2^. As a result, this implies that the donor-like states are mainly attributed to the electrical characteristics of IGZO TFT.

| 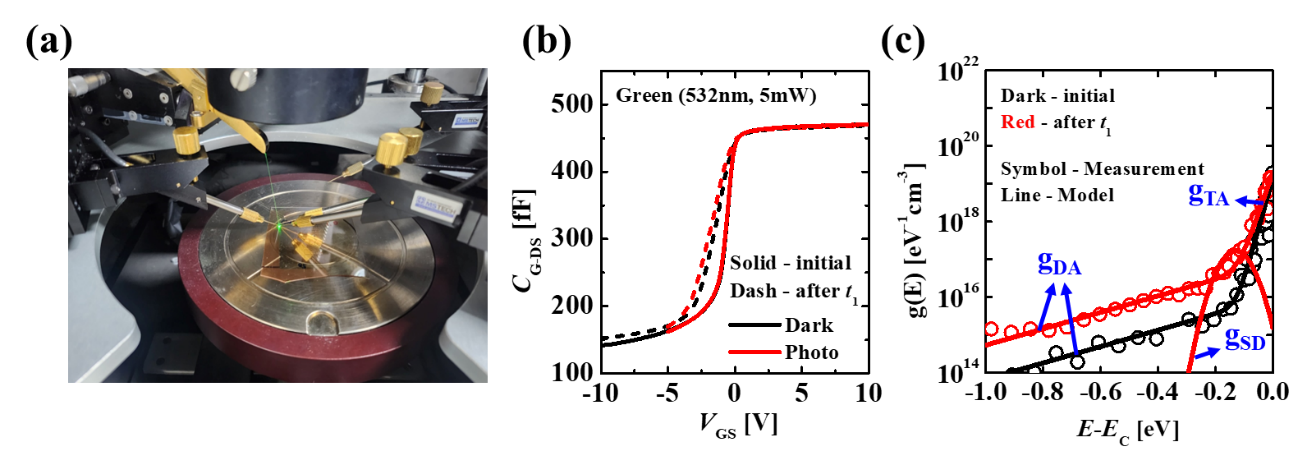 |
| --- |
| **Figure S1.** (a) A photograph for measuring the photo-response of the *CV* characteristic of IGZO TFT with 532 nm wavelength light. (b) The dark and photonic *CV* curves at initial state and after *t*_1_ (i.e., stress time = 20 sec). (c) Extracted subgap DOS distribution in the IGZO semiconductor before and after stress. |

**Table S1.** Subgap DOS parameters extracted from the IGZO TFTs before and after stress.

|  | **Initial** | **After stress** | **Unit** |
| --- | --- | --- | --- |
| **N_TA_ [×10^19^]** | 1.5 | 2.0 | [cm^-3^eV^-1^] |
| **kT_TA_** | 0.016 | 0.02 | [eV] |
| **N_DA_ [×10^16^]** | 1 | 8 | [cm^-3^eV^-1^] |
| **kT_DA_** | 0.2 | 0.2 | [eV] |
| **N_SD_ [×10^17^]** | - | 1.7 | [cm^-3^eV^-1^] |
| **kT_SD_** | - | 0.06 | [eV] |
| **E_SD_** | - | 0.13 | [eV] |
| **N_TD_ [×10^20^]** | 1 | 1 | [cm^-3^eV^-1^] |
| **kT_TD_** | 0.02 | 0.02 | [eV] |

**The images of IGZO TFT through the confocal thermal reflectance microscope.** Figure S2 shows the confocal thermal reflectance images of the IGZO TFT, which are measured by the Nanoscope systems CTRM700. These images show the thermal distribution of IGZO TFT before and after AC stress pulses. Figure S2 clearly indicates the increment of device temperature as the stress time increases. It can be explained by the Joule heating from the drive current since the IGZO TFT is composed of low thermal conductivity material (i.e., IGZO channel, glass substrate)^S3-S6^. Therefore, the short failure is confirmed not only by the electrical characteristics but also by the thermal images.

|  |
| --- |
| **Figure S2.** (a) The microscope image of IGZO TFT. The thermal reflectance images of IGZO TFT at (b) initial state and after (c) *t*_1_, (d) *t*_2_, (e) *t*_3_. |

**Gate leakage of IGZO TFT under AC stress.** Figure S3 shows the gate current (*I*_G_) under the AC stress with different *V*_low_. It clearly shows that the gate *I*_G_ is negligible compared to the drain current regardless of *V*_low_ (i.e., ~7 orders smaller). Therefore, the short failure is caused by the donor creation, not by the gate leakage current.

| 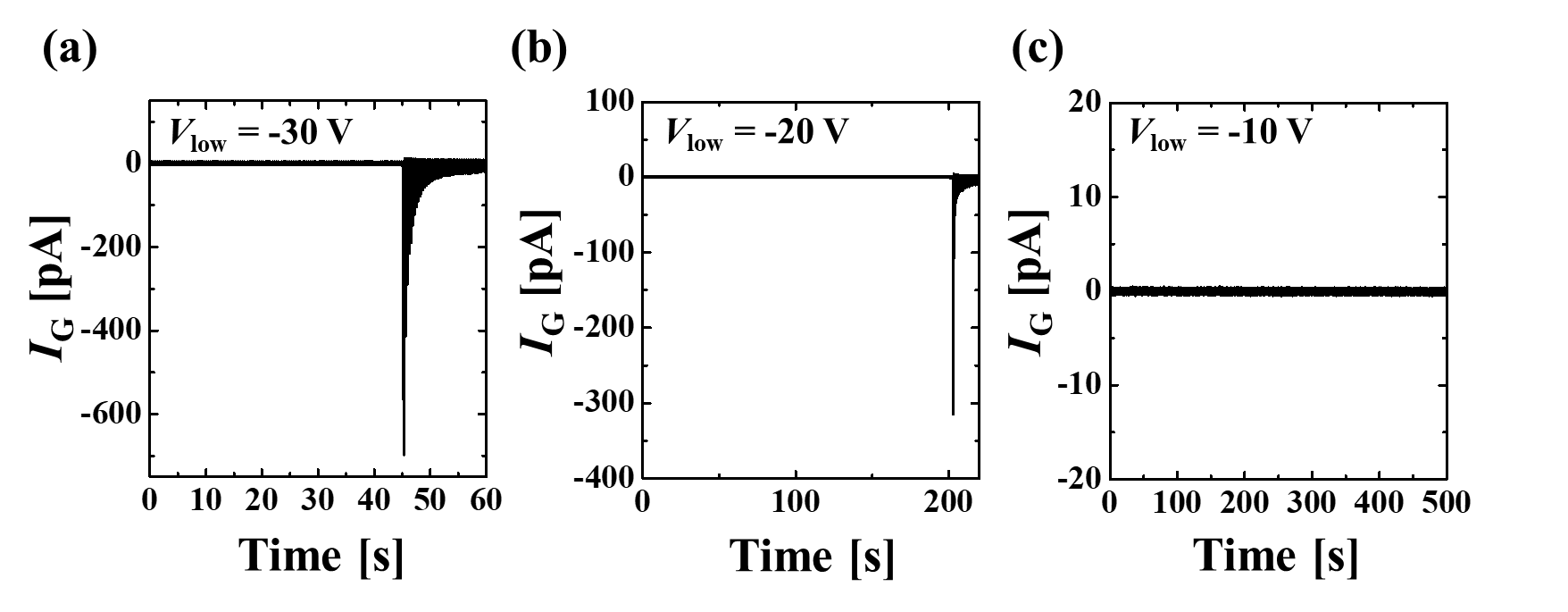 |
| --- |
| **Figure S3.** Transient response of gate current (*I*_G_) under the AC stress with *V*_high_ = 12.7 V and different *V*_low_; (a) *V*_low_ = -10 V, (b) *V*_low_ = -20 V, and (c) *V*_low_ = -30 V. These correspond to Figures 2a, 2c, and 2d. |

**Capacitance-voltage characteristics under the AC stress.** Figure S4 shows the capacitance-voltage (*CV*) curves with 100 kHz frequency before and after AC stress. It depicts that the *CV* curves shift toward a negative direction, the same as the result of Figure 2b as the stress increases. There are two noteworthy points in Figures S4b and S4c. First, there is a hump phenomenon after *t*_2_ in Figures S4b and S4c. These results represent that the donor creation generates near the drain side as well as the source side. Since the donor creation is located near the *E*_c_ (Figure S1c), the donor creation responds to the gate voltage (*V*_G_) faster than the electron accumulation at the channel. Therefore, the hump phenomenon occurs. Second, after the short failure (*t*_3_), the capacitance shows the same value regardless of the *C*_G-DS_, *C*_G-D_, and *C*_G-S_. This is because donor creation occurs in most channel regions (Figure 4d). As a result, the donor creation at the drain/source region and the short failure can be proved by the *CV* curves.

| 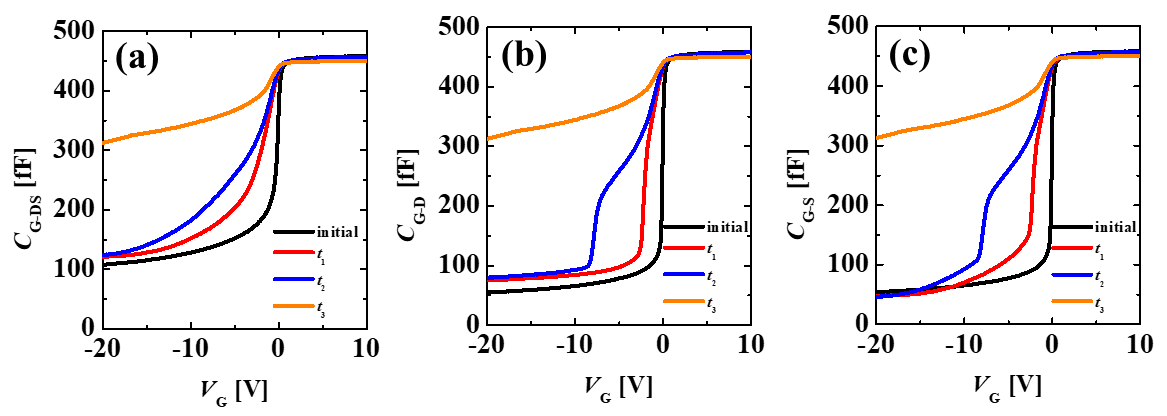 |
| --- |
| **Figure S4.** *CV* curves between (a) the gate-source/drain, (b) the gate-drain, and (c) the gate-source before and after AC stress pulses. |

**References (Supplementary information)**

S1. Bae H., Choi H., Jun S., Jo C., Kim Y. H., Hwang J. S., Ahn J., Oh S., Bae J.-U., Choi S.-J., Kim D. H., & Kim D. M. Single-Scan Monochromatic Phonic Capacitance-Voltage Technique for Extraction of Subgap DOS Over the Bandgap in Amorphous Semiconductor TFTs. *IEEE Electron Device Lett.* **34**, 1524–1526, DOI: https://doi.org/10.1109/LED.2013.2287511 (2013).

S2. Nahm H.-H., Kim Y.-S., & Kim D. H. Instability of amorphous oxide semiconductors via carrier-mediated structural transition between disorder and peroxide state. *Phys. Status Solidi B* **249**, 6, 1277–1281, DOI: https://doi.org/10.1002/pssb.201147557 (2012).

S3. Fujii M., Yano H., Hatayama T., Uraoka Y., Fuyuki T., Jung & and Kwon J.Y., Thermal analysis of degradation in Ga_2_O_3_−In_2_O_3_−ZnO thin-film transistors. *Jpn. J. Appl. Phys.* **47**, 8, 6236–6240, DOI: https://doi.org/10.1143/JJAP.47.6236 (2008).

S4. Kise K., Fujii M. N., Bermundo J. P., Ishikawa Y., & Uraoka Y. Self-heating suppressed structure of a-IGZO thin-film transistor. *IEEE Electron Device Lett.* **39**, 9, 1322–1325, DOI: https://doi.org/10.1109/LED.2018.2855152 (2018).

S5. Nguyen M.-C., On N., Ji H., Nguyen A. H.-T., Choi S., Cheon J., Yu K.-M., Cho S.-Y., Kim J., Kim S., Jeong J., & Choi R. Electrical characterization of the self-heating effect in oxide semiconductor thin-film transistors using pulse-based measurements. *IEEE Trans. Electron Devices* **65**, 6, 2492–2497, DOI: https://doi.org/10.1109/TED.2018.2826072 (2018).

S6. Lee D.-H., Jeong H.-S., Kim Y.-G., Kim M.-H., Son K. S., Lim J. H., Song S.-H., & Kwon H.-I. Quantitative Analysis of Channel Width Effects on Electrical Performance Degradation of Top-gate Self-aligned Coplanar IGZO Thin-film Transistors under Self-heating Stresses. *J. Semicond. Technol. Sci.* **23**, 1, 79–87, DOI: https://doi.org/10.5573/JSTS.2023.23.1.79 (2023).
